# The tongue of the red panda (*Ailurus fulgens fulgens* Cuvier, 1825)—a stereoscopy, light microscopy and ultrastructural analysis

**DOI:** 10.7717/peerj.12559

**Published:** 2021-11-25

**Authors:** Karolina Goździewska-Harłajczuk, Pavla Hamouzová, Joanna Klećkowska-Nawrot, Petr Čížek

**Affiliations:** 1 Department of Biostructure and Animal Physiology, Faculty of Veterinary Medicine, Wrocław University of Environmental and Life Sciences, Wrocław, Poland; 2 Department of Physiology, Faculty of Veterinary Medicine, University of Veterinary Sciences Brno, Brno, Czech Republic; 3 Department of Anatomy, Histology and Embryology, University of Veterinary Sciences Brno, Brno, Czech Republic

**Keywords:** Microstructure, Lingual papillae, Histology, Histochemistry, Scanning electron microscopy, Diet, *Ailurus fulgens* f., Highly selective forager

## Abstract

In the light of recent molecular studies, there are two phylogenetic species of the red panda (*Ailurus fulgens*): *Ailurus fulgens fulgens* and *Ailurus fulgens styani*. The red panda belongs to the endangered species living in the wild only in Asia and is included in the CITES list. Although the biology and diet of this species has been extensively described, the histological structure of the tongue and lingual glands has not yet been characterized in detail in relation to the lifestyle of this mammal under specific conditions and as a basis for comparative anatomical studies of the biodiversity of endemic species. Study samples were collected from two adult males of *Ailurus fulgens f*. held in Wrocław Zoological Garden. Both tongues were examined macroscopically; moreover, samples with lingual papillae for light microscopy and scanning electron microscopy (SEM) were collected from the apex, body and root of the tongue. Both tongues of the *Ailurus fulgens f*. males were approximately 9 cm long. The dorsal lingual surface was covered with mechanical and gustatory lingual papillae. Filiform papillae were observed on the apex and the body of the tongue, while small conical papillae were observed on the root of the tongue. An elongated, 1–1.5 cm long cylinder-shaped lyssa was observed in the ventral part of the apex. Moreover, most numerous and largest round in shape fungiform papillae were observed on the apex and on the border of the body and root of the tongue, located directly rostrally to 12–13 round and oval in shape vallate papillae. The SEM study showed that filiform papillae on the apex had several long secondary processes, while filiform papillae on the body of the tongue were taller and their secondary papillae were shorter than the equivalent structures on the apex of the tongue. The SEM study showed numerous taste pores on the surface of the fungiform papilla, while irregular surface of the vallate papillae, however some of them had smoother surface. Mixed glands (comprised of mucous acini and serous acini) were present within the vallum (within the connective tissue core) of the vallate papilla. Beneath the papillae more serous glands were observed, while the posterior lingual glands in the caudal part of the root of the tongue were mucoserous (mucous units were prevalent). A characteristic feature of the tongue of *Ailurus fulgens f*. was the presence of lyssa, which is comparable to other representatives of Carnivora, but the number of vallate papillae was individually variable. The lack of strongly developed mechanical conical papillae probably may be related to the type of plant food that is particularly dominant in red panda. Further differences between *Ailurus fulgens f*. and *Ailurus fulgens s*. cannot be excluded. The results of these studies may be useful especially for veterinarians specializing in working with exotic animals and people dealing with wildlife conservation.

## Introduction

The red panda (*Ailurus fulgens* Cuvier, 1825) belongs to the mammal Ailuridae family in the Carnivora order (Fisher et al., 2008; Fisher, 2011; Glatston et al., 2015; Makungu, 2015). Molecular analyses have confirmed that there are two phylogenetically distinct species of red panda, namely *Ailurus fulgens fulgens* (Himalayan red panda) and the other species *Ailurus fulgens styani* (Chinese red panda) (Groves, 2011, Hu et al., 2017, 2020). The two phylogenetically distinct species of red panda live in Asia, in different geographic areas (Groves, 2011; Ghose & Dutta, 2011; Hu et al., 2011, 2017, 2020; Zhou et al., 2013; Dangol, 2014; Dorjee, Chakraborty & Dutta, 2014; Sharma, Belant & Swenson, 2014).

According to the Red List of Threatened Species of IUCN, the red panda is an endangered species (EN). It is also included in Appendix 1 of the Convention on International Trade in Endangered Species of Wild Fauna and Flora (CITES). In Nepal, the red panda is legally protected by National Parks and Wildlife Conservation Act (1973, http://cfpcc.gov.np/downloadfile/National-Parks-and-Wildlife-Conservation-Act-2029-1973_english_1517477214.pdf). In English, the red panda (*Ailurus fulgens*) is also called “lesser panda” or “red cat-bear”.

The studies to date in the biology of red panda and its diet, in particular, show that despite being a member of the Caniformia suborder, it feeds on leaves, mainly young leaves and shoots of bamboo (Pradhan, Saha & Khan, 2001; Wei & Zhang, 2011a, 2011b; Panthi et al., 2012). Furthermore, the type of tree leaves, shrubs, herb species and moss in the diet of red panda may vary also according to the season and region where individuals of this species occur (Roberts & Gittleman, 1984; Gittleman, 1993; Panthi et al., 2012, 2015; Nijboer & Dierenfeld, 2011; Thapa & Basnet, 2015). It is necessary to take into account variations in the diet of red panda individuals held in captivity in zoological gardens around the world. Interestingly, individuals living in the natural habitat of red panda have specific distinct microbiota of gastrointestinal tract, contrary to other members of the Carnivora order (Zeng et al., 2018). Specialised anatomical structure of the digestive tract includes not only secretomotor activity of stomach, secretory activity of digestive glands and secretomotor activity of intestines, but also initial treatment of food in oral cavity. One of the fundamental anatomical structures taking part in the complex process of initial treatment of food, apart from lips, teeth, palate and salivary glands, is the tongue (Iwasaki, 2002; Iwasaki et al., 2019).

Studies in microstructure of the lingual surface were carried out in many representatives of the Carnivora order, both in domestic species, such as cat (Boshell, Wilborn & Singh, 1982; Haddad et al., 2019; Kobayashi et al., 1988; Ojima et al., 1997, 2000) and dog, and in non-domestic species (held in zoological gardens or living in natural habitats). As the Carnivora order is a large group, studies in tongue were carried out in both suborders, namely the Feliformia and Caniformia.

For non-domestic species in the Feliformia suborder, lingual surface was studied (macroscopic analysis, type of lingual papillae with the use of light microscopy, scanning electron microscopy or transmission electron microscopy techniques), in the following members of the Felidae family, among others: the tiger *Panthera tigris* (Emura et al., 2004), lion *Panthera leo* (Toprak & Ulusoy, 2011), jaguar *Panthera onca* (Emura, Okumura & Chen, 2013), fishing cat *Prionailurus viverrinus* (Emura, Okumura & Chen, 2014, Bengal tiger *Panthera tigris tigris* (Kim et al., 2014), Persian leopard *Panthera pardus saxicolor* (Sadeghinezhad et al., 2017), puma *Puma concolor* (Erdoğan et al., 2018), Asian golden cat *Catopuma temminckii* (Emura, 2018b), leopard *Panthera pardus* (Emura, 2018a), ocelot *Leopardus pardalis* (Freire et al., 2019); it was also studied in the Herpestidae family: in mongoose (Iwasaki & Miyata, 1990). For the Caniformia suborder, microstructure of the tongue was studied in some members of the Canidae family, for instance in the bush dog *Speothos venaticus* (Emura et al., 2000), racoon dog *Nyctereutes procyonoides* and fox (Emura et al., 2006), black-backed jackal *Canis mesomelas* (Emura & Sugiyama, 2014), African wild dog *Lycaon pictus* (Goździewska-Harłajczuk, 2018), wolf *Canis lupus* (Haligur, Ozkadif & Alan, 2019), family Ursidae: Asian black bear *Ursus thibetanus* (Emura et al., 2001, Pastor et al., 2011), American black bear *Ursus americanus*, spectacled bear *Tremarctos ornatus*, Malayan sun bear *Helarctos malayanus* (Pastor et al., 2011), giant panda *Ailuropoda melanoleuca* (Pastor, Barbosa & De Paz, 2008), polar bear *Ursus maritimus* (Emura, Sugiyama & Kusuda, 2017), family Mustelidae: Japanese marten *Martes melampus* (Emura, Okumura & Chen, 2007), Japanese badgers *Meles meles anakuma* (Yoshimura, Shindo & Kageyama, 2009), ferret *Mustela putorius furo* (Takemura et al., 2009), American mink *Neovison vison* (Yoshimura et al., 2014), least weasel *Mustela nivalis* (El-Bakary & Emura, 2016), Asian small-clawed otter *Aonyx cinereus* (Emura & Sugiyama, 2016), family Procyonidae: crab-eating raccoon *Procyon cancrivorus* (Correa et al., 2012), common raccoon *Procyon lotor* (Miyawaki et al., 2020), family Phocidae: Spotted seal *Phoca largha* (Yoshimura et al., 2007), and also in members of the Otariidae family, such as the California sea lion *Zalophus californianus californianus* (Yoshimura, Shindoh & Kobayashi, 2002). Moreover, the anatomic structure of lyssa, a specific structure in the tongue, was studied in detail in dogs and cats (Besoluk, Eken & Sur, 2006; Shoeib, Rizk & Hassanin, 2014; Sultana et al., 2017), Persian leopard (Sadeghinezhad et al., 2017) or crab-eating racoon (Correa et al., 2012).

Although the lingual surface in the lesser panda *Ailurus fulgens f*. was analysed using SEM (Emura, Okumura & Chen, 2009), no histological studies of the lingual surface have been carried out to date, nor have the properties of the secretions of the lingual glands been analyzed in this species *Ailurus fulgens f*. Therefore, the aim of the present study is to provide a detailed description of the individual papillae present in the lingual surface and to characterize the lingual glands using SEM and light microscopy. Further, the aim of the present study is to compare features of the tongue typical for the red panda to other members of the Carnivora order, especially with regard to the presence and structure of lyssa, from the point of view of a surgical study. Additionally, the results of the present study will form a basis for comparative anatomical studies of the biodiversity of endemic species.

## Materials & methods

### Collection of the specimens

The specimens were gathered in the collection of the Division of Animal Anatomy in 2020–2021. The tongues (S1) were collected from two adult males of the red panda (*Ailurus fulgens f*.) held in Wrocław Zoological Garden. The post mortem examination of the animals revealed no pathologies of the oral cavity. Registered permissions for the post-mortem collection of specimens was issued by the District Veterinary Officer in Wroclaw (No. PIW Wroc. UT-45/5/16, No. PIW Wroc. UT-45/6/16, No. PIW Wroc. UT-45/8/16). The tongues were rinsed with physiological saline solution. The length and width of the tongues were recorded and the lyssas were prepared. An electronic caliper (with an accuracy of 0.1 mm) was used to take the measurements and a Canon EOS 300 camera was used to document the analysis.

### Stereoscopic analysis

Individual types of lingual papillae (visualization of the dorsal surface of the tongue and visualization of individual lingual papillae in longitudinal cross section of the tongue, visualization of ventral surface of the tongue) and lyssa were examined using a Zeiss Stemi 2000-C microscope (Carl Zeiss, Jena, Germany) and were photographed subsequently.

### Light microscopy–histological and histochemical study

Samples for histological and histochemical studies (visualization of transverse cross section of the entire tongue, lyssa, longitudinal cross section of the lingual papillae and visualization of the posterior lingual glands) were taken from the apex, body and root of the tongue. Sections for histological studies included filiform papillae (from the apex and body of the tongue), small conical papillae (from the root of the tongue), fungiform papillae (from the apex and body), vallate papillae and samples of the tongue in transverse cross section from the apex of the tongue including the lyssa. Additional sections were taken from the root of the tongue for histochemical studies. The specimens were fixed in 4% buffered formaldehyde (Chempur, Poland). Next, they were dehydrated in a series of alcohol dilutions, cleared in xylene and impregnated with paraffin. The specimens were cut using a Slide 2003 (Pfm AG, Köln, Germany) sliding microtome.

Subsequently, the specimens were subjected to histochemical studies and were stained with hematoxylin&eosin (H&E) (to assess the general structure of studied tissue) and additionally with Masson-Goldner trichrome and Azan trichrome (to assess connective tissue and epithelium), whereas samples for histochemical studies (Spicer & Henson, 1967) of the lingual glands (to assess the type of secretions of these lingual glands) were stained with periodic acid-Schiff (PAS), alcian blue pH 1.0 (AB pH 1.0), alcian blue pH 2.5 (AB pH 2.5—to prove glycosaminoglycan in mucins), periodic acid-Schiff-alcian blue pH 2.5 (PAS-AB pH 2.5) and Hale’s dialysed iron staining (HDI). The slides were analysed using Zeiss Axio Scope A1 light microscope (Carl Zeiss, Jena, Germany). Histological measurements of individual lingual papillae were carried out using the Axio Vision 4.8 (Carl Zeiss MicroImaging GmbH, Jena, Germany) software. Histological examination results were presented as mean and standard deviation (SD).

### Scanning electron microscopy

Samples for SEM examination were collected from the apex, body and root of the tongue. Filiform papillae, small conical papillae, fungiform papillae, vallate papillae and the caudal surface of the root of the tongue without the papillae were analysed in the SEM study. The collected samples were fixed with 2.0% glutaraldehyde dissolved in 0.1 M phosphate buffer at pH 7.4 in small test tubes. The samples were then prepared for SEM analysis using Čížek et al. (2020). Further, the tissue samples were dried at critical point (CPD 030 Critical Point Dryer; Bal-Tec, Los Angeles, CA, USA) and then gold-coated (Balzers SCD 040 by current 30 mA for 4 min). SEM photographs were taken using Tescan VEGA TS 5,136 XM (s.r.o.; Tescan, Brno, Czech Republic) scanning electron microscope in a high vacuum and accelerated voltage 20 kV using an SE detector. The surface of individual lingual papillae, presence or absence of taste pores of the papillae, presence and size of openings of the posterior lingual glands and the number and length of secondary processes of the filiform papillae were assessed in the SEM study.

The detailed results of the analysis of the tongue structure in *Ailurus fulgens f*. in the current study were described using the terms from the International Committee on Veterinary Gross Anatomical Nomenclature (2017) and the International Committee on Veterinary Histological Nomenclature (2017).

## Results

### Macroscopic analysis of the lingual surface and lyssa

**Figure 1:**
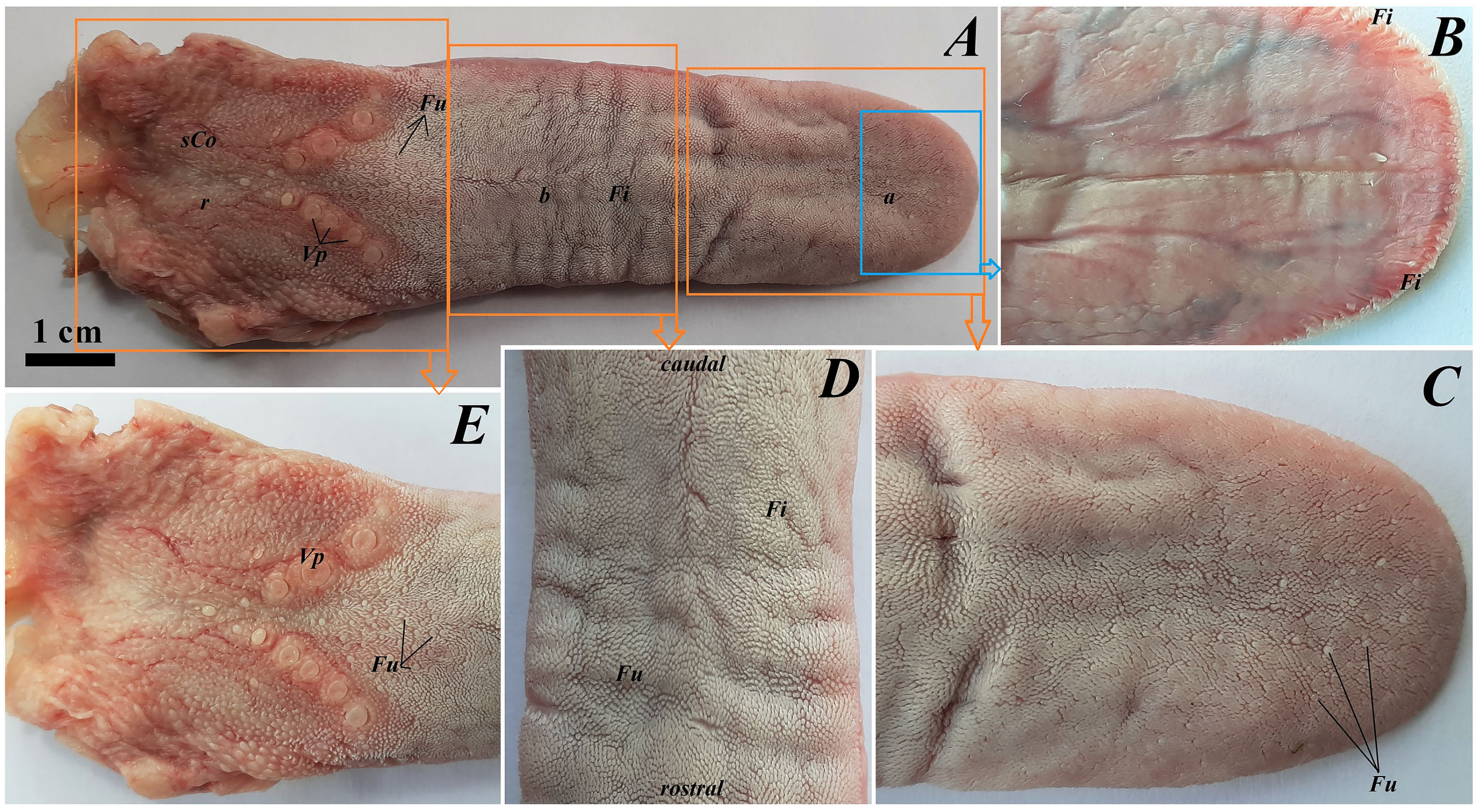
Photograph of the tongue of the red panda (*Ailurus fulgens f*.). (A) Dorsal surface of the tongue, Bar = 1 cm. (B) Ventral surface of the apex of the tongue. (C) Magnification of the dorsal surface of the apex with numerous fungiform papillae and dominant filiform papilae. (D) Magnification of the dorsal surface of the body of the tongue. (E) Magnification of the dorsal surface of the root of the tongue. Abbreviations: a, apex of the tongue; b, body of the tongue; caudal, caudal orientation of the tongue; Fi, filiform papilla; Fu, fungiform papilla;r, root of the tongue; rostral, rostral orientation of the tongue; *sCo*, small conical papilla; Vp, vallate papilla.

**Figure 2:**
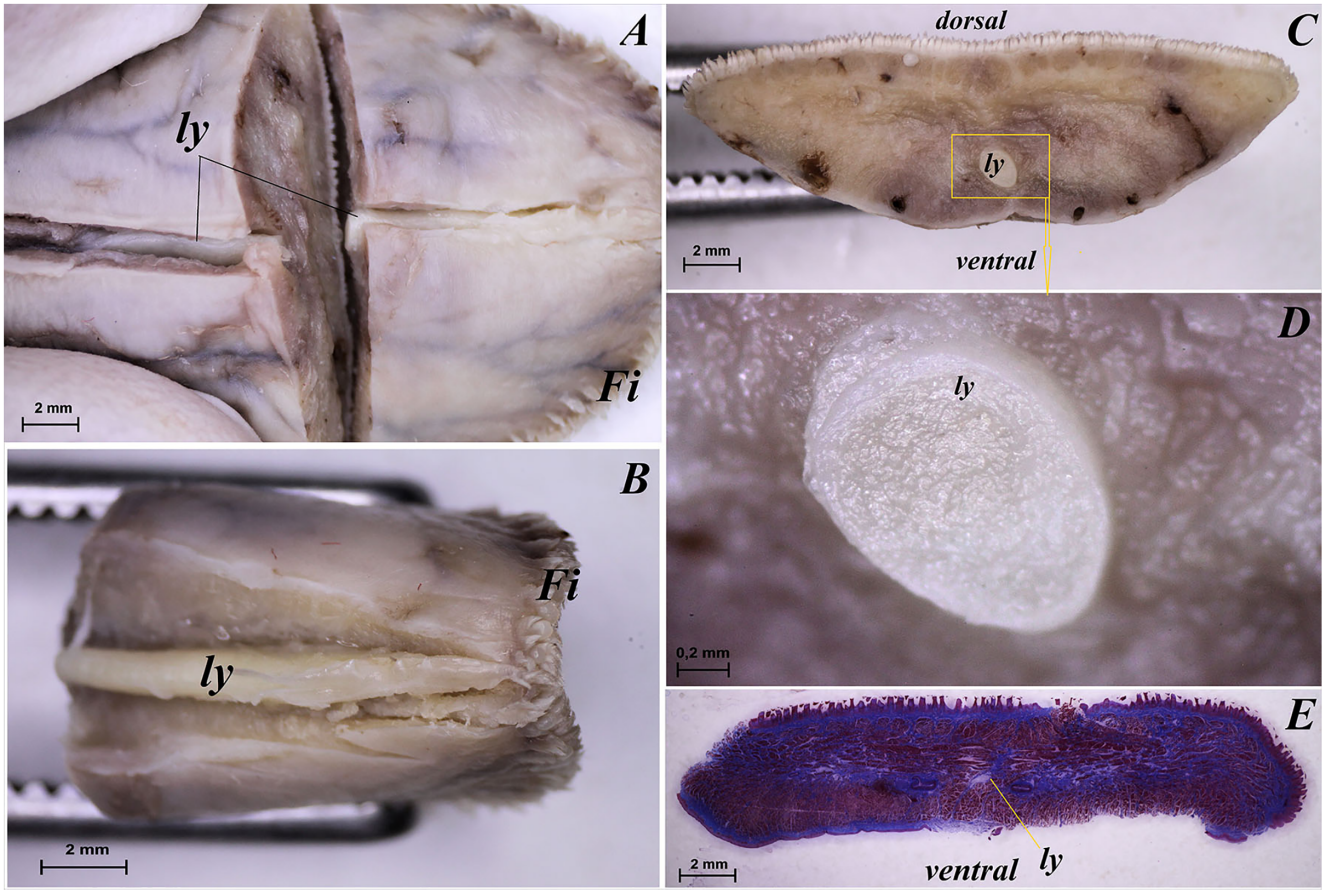
Stereoscopic and histological analysis of the lyssa on the ventral surface of the tongue of the red panda (*Ailurus fulgens f*.). (A) Transverse cross section of the lyssa. (B) Magnification of the rostral part of the lyssa. (C) Transverse cross section of the apex of the tongue with well visible location of the lyssa. (D) Magnification of the oval in shape lyssa (the rostral part of the lyssa). (E) Histomicrograph of the transverse cross section of the tongue tongue with caudal part of the lyssa. Bar = 2 mm (A–C, E); Bar = 0.2 mm (D). Abbreviations: dorsal, dorsal surface of the tongue; Fi, filiform papilla; ly, lyssa; ventral, ventral surface of the tongue.

The tongue of *Ailurus fulgens f*. was approximately 9 cm long. It was composed of a rounded apex, body and root (Figs. 1A–1E). The widths were 1.5 cm, 2.5 cm and 3.2 cm in the apex, body and root, respectively. The thicknesses were 0.4 cm, 0.4 cm and 1.1 cm in the apex, body and root, respectively. The dorsal surface of the tongue was not pigmented and no median groove was observed in it (Fig. 1A). The dorsal surface was covered with mechanical lingual papillae and gustatory lingual papillae (Figs. 1A, 1C–1E). The first group, *i.e*. mechanical papillae, were filiform papillae, which were observed on the apex and body of the tongue (Figs. 1C and 1D), and small conical papillae, which were observed on the root of the tongue (Fig. 1E). The second group, *i.e*. gustatory papillae, included fungiform papillae and vallate papillae. The most numerous of the gustatory papillae were round in shape fungiform papillae and the largest of them were located on the apex, with the density of 63/cm^2^, and at the border of the body and root of the tongue (in directly rostral location to vallate papillae), with the density of 59/cm^2^ (Figs. 1A, 1C, and 1E). 12–13 vallate papillae were observed (Figs. 1A and 1E). The majority of vallate papillae were round in shape, while some of them were oval (Fig. 1E). An annular pad was observed around each vallate papilla. The ventral surface of the tongue was smooth (Fig. 1B). Furthermore, an elongated, 1–1.5 cm long cylinder-shaped lyssa was observed in the ventral part of the apex. The shape of the lyssa was oval in the transverse cross section (Figs. 2C and 2D).

### Histological and ultrastructural analysis of the lingual surface

#### Apex of the tongue

**Figure 3:**
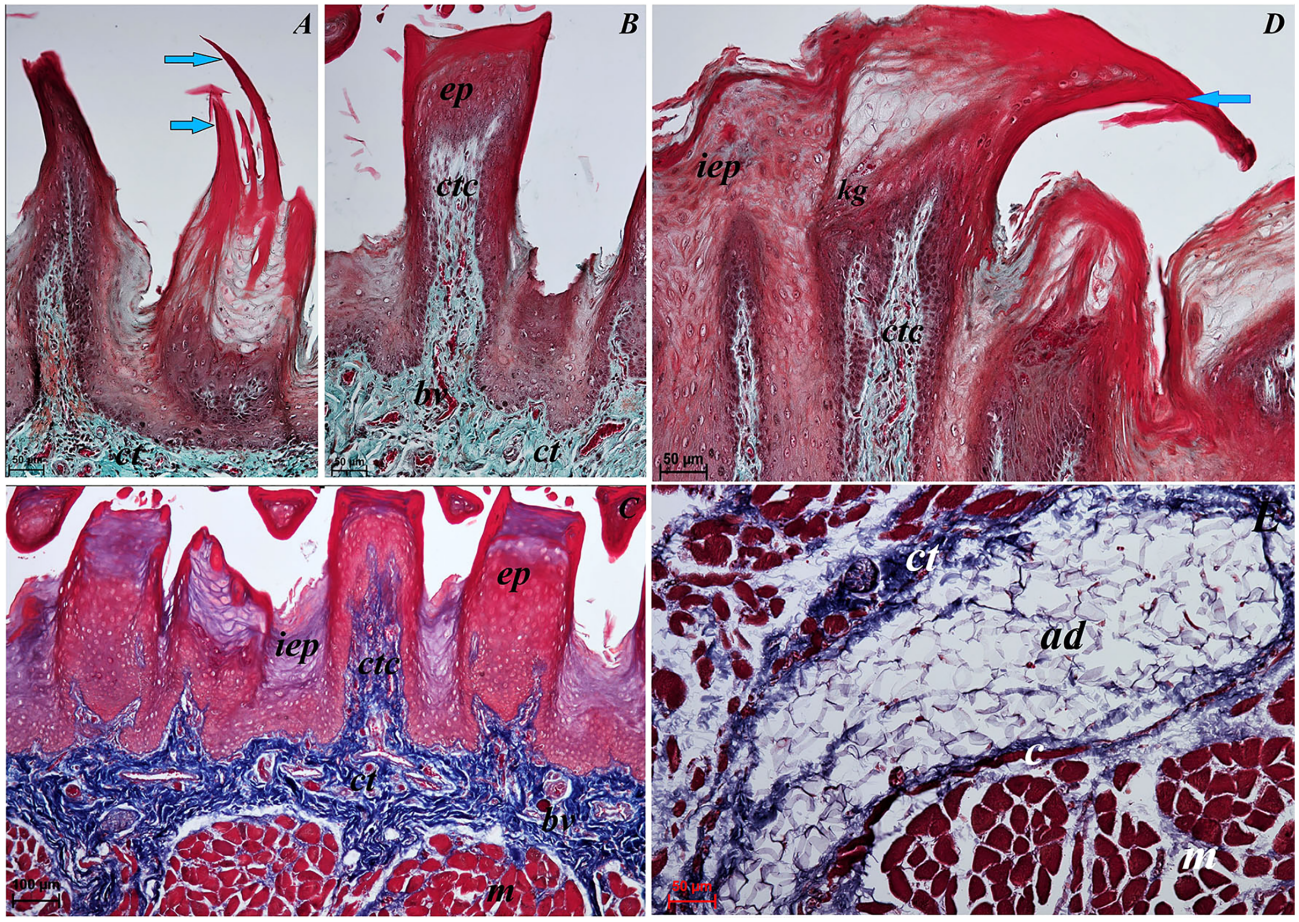
Histological analysis of the filiform papillae and lyssa of the tongue of the of red panda (*Ailurus fulgens f*.). (A) Two filiform papilla from apex of the tongue. See several processes (*stratum corneum*) of the keratinized stratified sqamous epithelium–blue arrows. Masson-Goldner trichrome staining. (B) Filiform papilla from body of the tongue - transverse cross section of the papilla. Masson-Goldner trichrome staining. (C) Four filiform papillae from body of the tongue with well defined interpapillary epithelium. Azan trichrome staining. (D) Magnification of the filiform papilla from body of the tongue - longitudinal cross section of the papilla. See not numerous keratohyaline granules and well visible *stratum corneum* of the keratinized stratified squamous epithelium (blue arrow). Masson-Goldner trichrome staining. (E) Magnification of the lyssa in the caudal part. Azan trichrome staining. Bar = 50 µm (A, B, D, E); Bar = 100 µm (C). Abbreviations: ad, adipose cells of the lyssa; bv, blood vessel; c, capsule of the lyssa; ct, connective tissue; ctc, connective tissue core; iep, interpapillary epithelium; kg, keratohyaline granules; m, muscle fibers.

**Figure 4:**
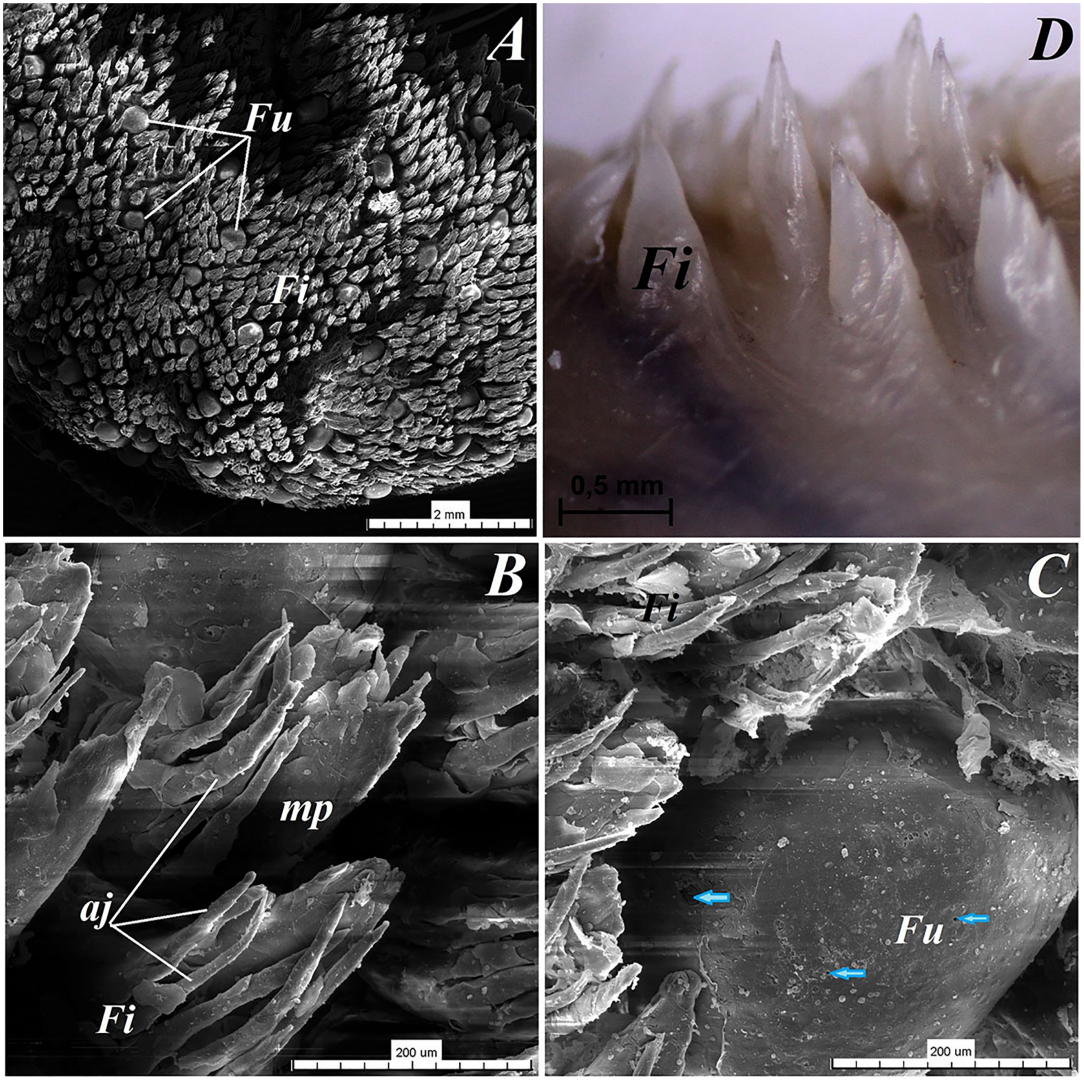
Stereoscopic and SEM analysis of the apex of the tongue of the red panda (*Ailurus fulgens f*.). (A) Dorsal surface of the apex of the tongue with numerous filiform papillae and round in shape fungiform papillae–SEM analysis. (B) Magnification of the filiform papilla with several (6–8) secondary processes with different length–SEM analysis. See that the cone of the main papilla is also divided. (C) Magnification of the fungiform papilla from apex of the tongue–SEM analysis. See several taste pores (blue arrow) on the surface of the fungiform papilla. (D) Magnification of the filiform papilla from lateral margin of the apex of the tongue–stereoscopic analysis. See that the cone of main papilla has several spines. Bar = 2 mm (A); Bar = 200 µm (B,C); Bar = 0.5 mm (D). Abbreviations: aj, secondary processes. Fi, filiform papilla. Fu, fungiform papilla. mp, main part of the filiform papilla.

The dorsal surface of the apex of the tongue in *Ailurus fulgens f*. was covered with keratinized stratified squamous epithelium (Fig. 3A). Filiform papillae (Fig. 4A) and less numerous fungiform papillae (Fig. 4A) were observed on the dorsal surface of the apex of the tongue.

The light microscopy analysis showed that the filiform papillae on the apex were composed of connective tissue core and stratified epithelium with well defined *stratum corneum* (Fig. 3A). The SEM study showed presence of 6–8 secondary projections within each filiform papilla (on the anterior central surface of the main papilla) (Fig. 4B). The secondary projections were thin and long, while the main cones of filiform papillae were subdivided too (Fig. 4B). The filiform papillae on the lateral margin of the apex of the tongue had divided cones of main papillae and these cones had several spines (Fig. 4D). In SEM the fungiform papillae from apex of the tongue had round shape (Figs. 4A and 4C). On the surface of the fungiform papillae the openings (fewer than 10) of the taste buds were recognized (Fig. 4C). The height of fungiform papillae was 875.14 ± 87.67 µm, while the width was 398.57 ± 142.95 µm.

The transverse cross section in the histological study showed lyssa in the ventral part of the apex of the tongue (Fig. 1E). The lyssa was surrounded by a thin-walled connective tissue capsule, in which muscle fibers were observed. Inside the lyssa, numerous adipose cells were revealed (Fig. 3E).

#### Body of the tongue

**Figure 5:**
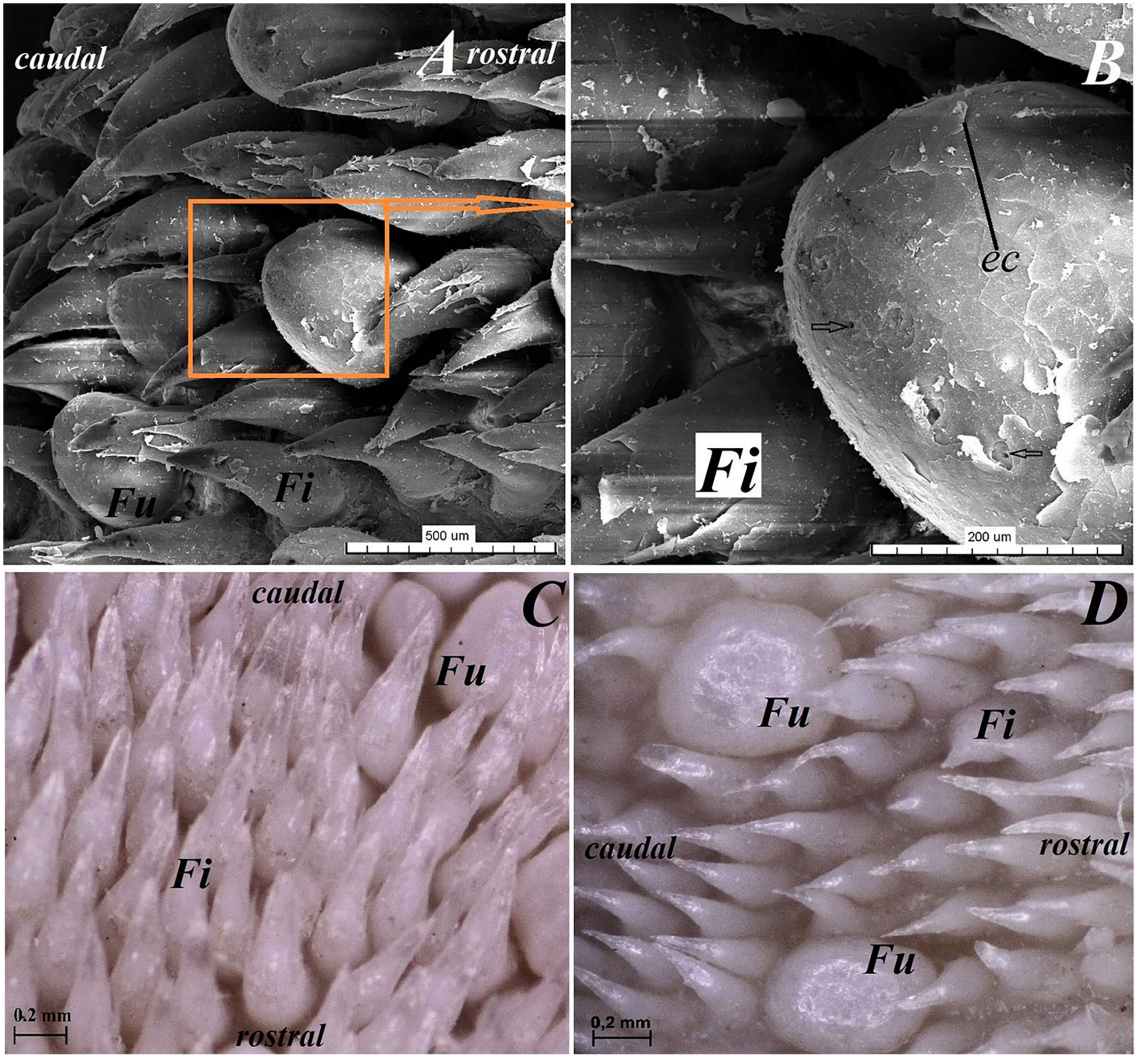
Stereoscopic and SEM analysis of the body of the tongue of the red panda (*Ailurus fulgens f*.). (A) Four fungiform papillae within the numerous filiform papillae–SEM analysis. (B) Magnification of the round in shape fungiform papilla with several taste pores on the dorsal surface of this papilla–black arrow-SEM analysis. (C) Filiform papillae and one fungiform papilla from body of the tongue. See the several secondary processes of the filiform papillae–stereoscopic analysis. (D) Numerous filiform papillae from caudal dorsal surface of the body of the tongue (directly rostral to the vallate papillae)-stereoscopic analysis. See two big fungiform papillae. Bar = 500 µm (A); Bar = 200 µm (B); Bar = 0.2 mm (C and D). Abbreviations: caudal, caudal orientation of the tongue; ec, exfoliated cell; Fi, filiform papilla; Fu, fungiform papilla; rostral, rostral orientation of the tongue.

**Figure 6:**
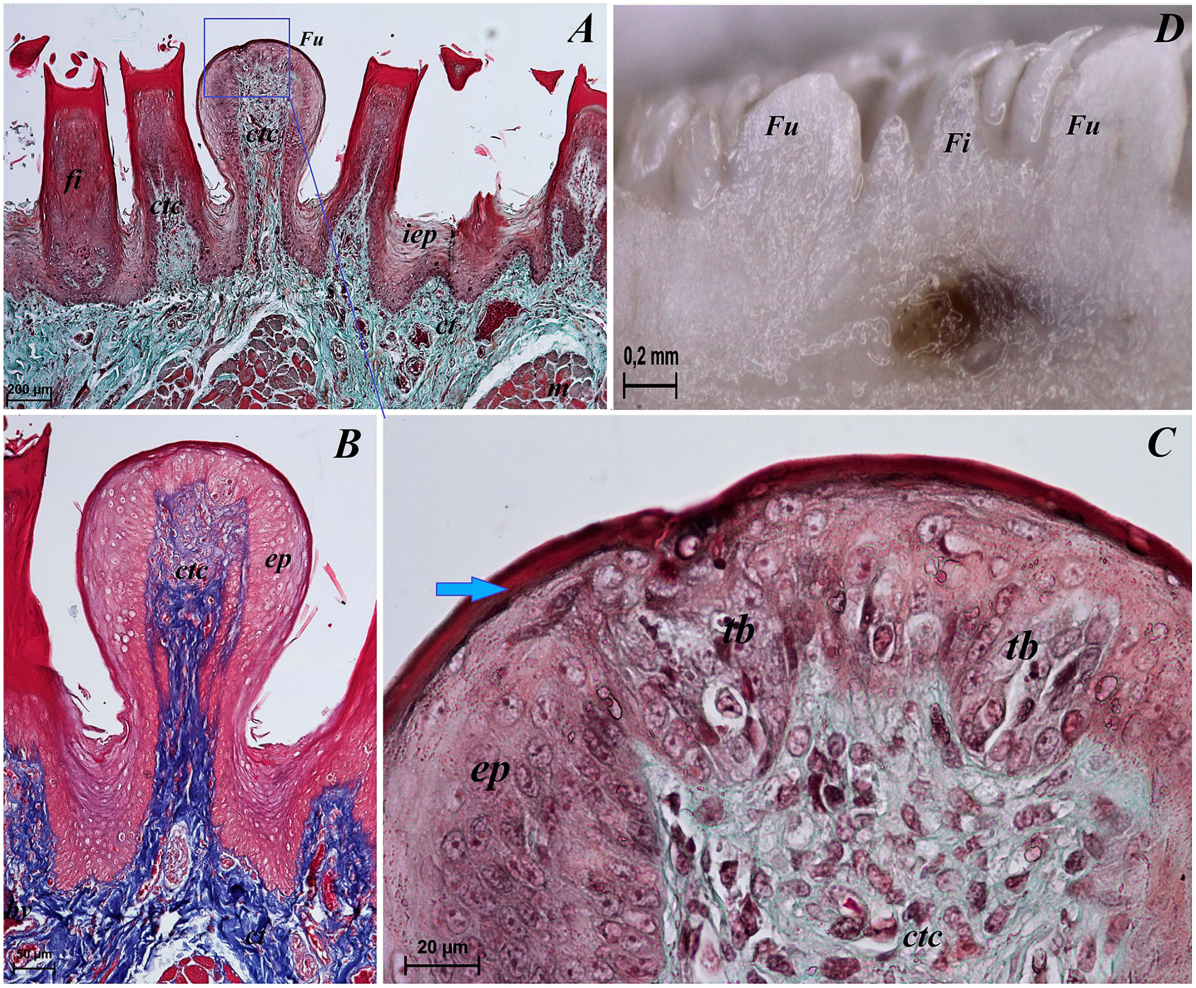
Sstereoscopic and histological analysis of the fungiform papillae of the tongue of the red panda (*Ailurus fulgens f*.). (A) Single fungiform papilla between four filiform papillae–see the elongate shape of the connective tissue core of the fungiform papilla. Masson-Goldner trichrome staining. (B) Magnification of the single fungiform papilla. Azan trichrome staining. (C) Magnification of the dorsal surface of the fungiform papillae with two taste buds within the papilary epithelium. See a thin superficial layer (*stratum corneum*) of the keratinized stratified squamous epithelium–blue arrow. Masson-Goldner trichrome staining. (D) Two fungiform papillae between filiform papillae–longitudinal cross section of the body of the tongue–stereoscopic analysis. Bar = 200 µm (A); Bar = 50 µm (B); Bar = 20 µm (C); Bar = 0.2 mm (D). Abbreviations: ctc, connective tissue core; ep, epithelium; Fi, filiform papilla; Fu, fungiform papilla; iep, interpapillary epithelium; tb, taste bud.

The dorsal surface of the body of the tongue of *Ailurus fulgens f*. was covered with keratinized stratified squamous epithelium (Figs. 3B–3D). Filiform papillae (Figs. 5A and 5B) and predominantly round in shape small fungiform papillae (Figs. 5A and 5B) were observed on the dorsal surface of the body of the tongue. *Stratum corneum* of the epithelium within the filiform papillae was well defined (Figs. 4C and 4D). Each filiform papilla was separated from one another by interpapillary epithelium (Figs. 4C and 4D). The SEM study showed that filiform papillae on the body of the tongue had bigger main papillae and shorter and fewer secondary projections (Fig. 5A), or secondary projections were absent. By comparison to the filiform papillae, the stratum corneum of the stratified squamous epithelium of fungiform papillae formed a considerably thinner layer (Figs. 6A–6C). Furthermore, elongated taste buds were observed within the epithelium of fungiform papillae (Figs. 6B and 6C), located predominantly in the dorsal part of this epithelium. The SEM analysis of the fungiform papillae confirmed the presence of the opening of the taste buds (Fig. 5B).

#### Root of the tongue

**Figure 7:**
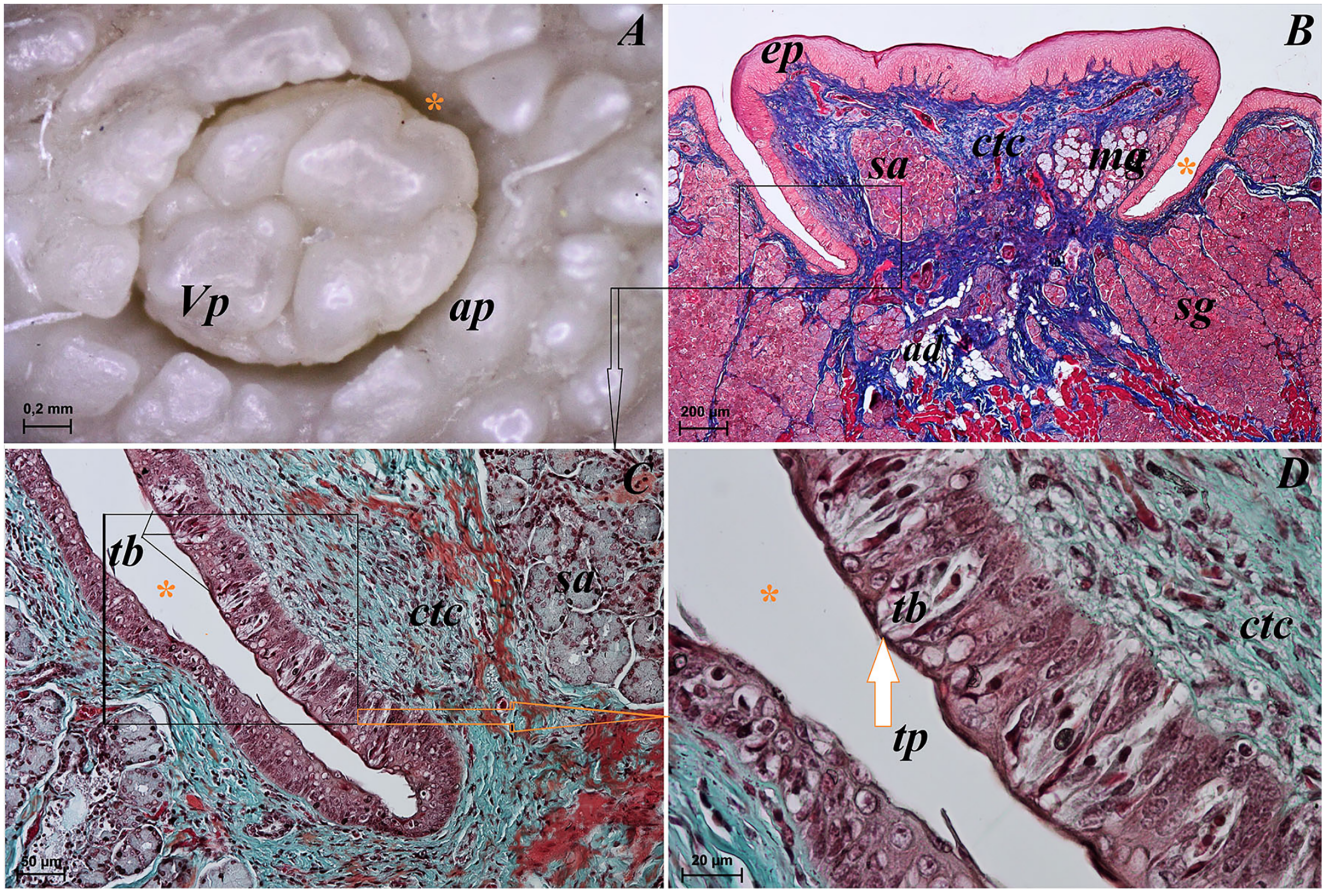
Stereoscopic and histological analysis of the vallate papillae of the tongue of the red panda (*Ailurus fulgens f*.). (A) Single vallate papilla with irregular dorsal surface and with prominent papillary groove and annular pad around of the papilla–stereoscopic analysis. (B) Vallum of the papilla with well defined mixed glands within the connective tissue core of the papilla. See the prominent papillary groove (yellow asterisk). Azan trichrome staining. (C) Magnification of the lateral wall of the vallate papilla with several taste buds. Masson-Goldner trichrome staining. (D) Magnification of the elongate taste buds with their taste pores. Masson-Goldner trichrome staining. Bar = 0.2 mm (A); Bar = 200 µm (B); Bar = 50 µm (C); Bar = 20 µm (D). Abbreviations: ap, annular pad; ad, adipose cells; ctc, connective tissue core; ep, epithelium; mg, mucoserous glands; sa, serous acini; sg, serous glands; tb, taste bud; tp, taste pore; Vp, vallate papilla. * yellow asterisk, groove of the vallate papilla.

The dorsal surface of the root of the tongue in *Ailurus fulgens f*. was covered with keratinized stratified squamous epithelium. Between the body and the root of the tongue, vallate papillae were revealed, which were characterized by the presence of numerous taste buds, located in the lateral wall of the vallum of these gustatory papillae (Figs. 7B–7D).

**Figure 8:**
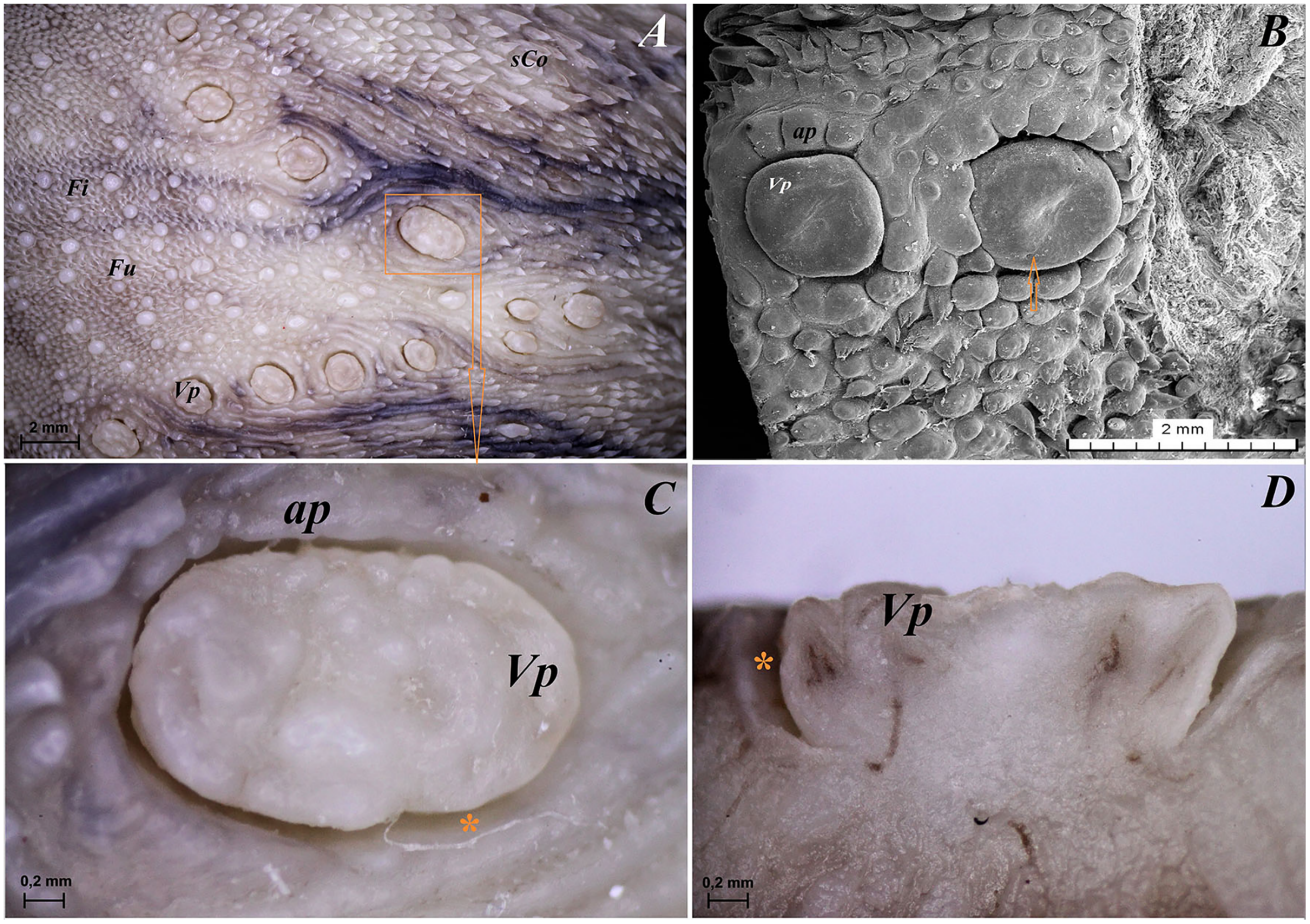
Stereoscopic and SEM analysis of the vallate papillae of red panda (*Ailurus fulgens f*.). (A) Dorsal surface of the root of the tongue with all 13 vallate papillae of male 2 of red panda. (B) Microstructure of the two vallate papillae–SEM analysis. (C) Magnification of the biggest oval in shape valate papilla with clear papillary groove around of the papilla. See the irregular dorsal surface of the vallum of the papilla. (D) Longitudinal cross section of the vallum of papilla with clear and deep papillary groove. Bar = 2 mm (A and B); Bar = 0.2 mm (C and D). Abbreviations: ap, annular pad with irregular surface; Fi, filiform papilla; Fu, fungiform papila; sCo, small conical papilla; Vp, vallate papilla; *yellow asterisk, groove of the vallate papilla.

**Figure 9:**
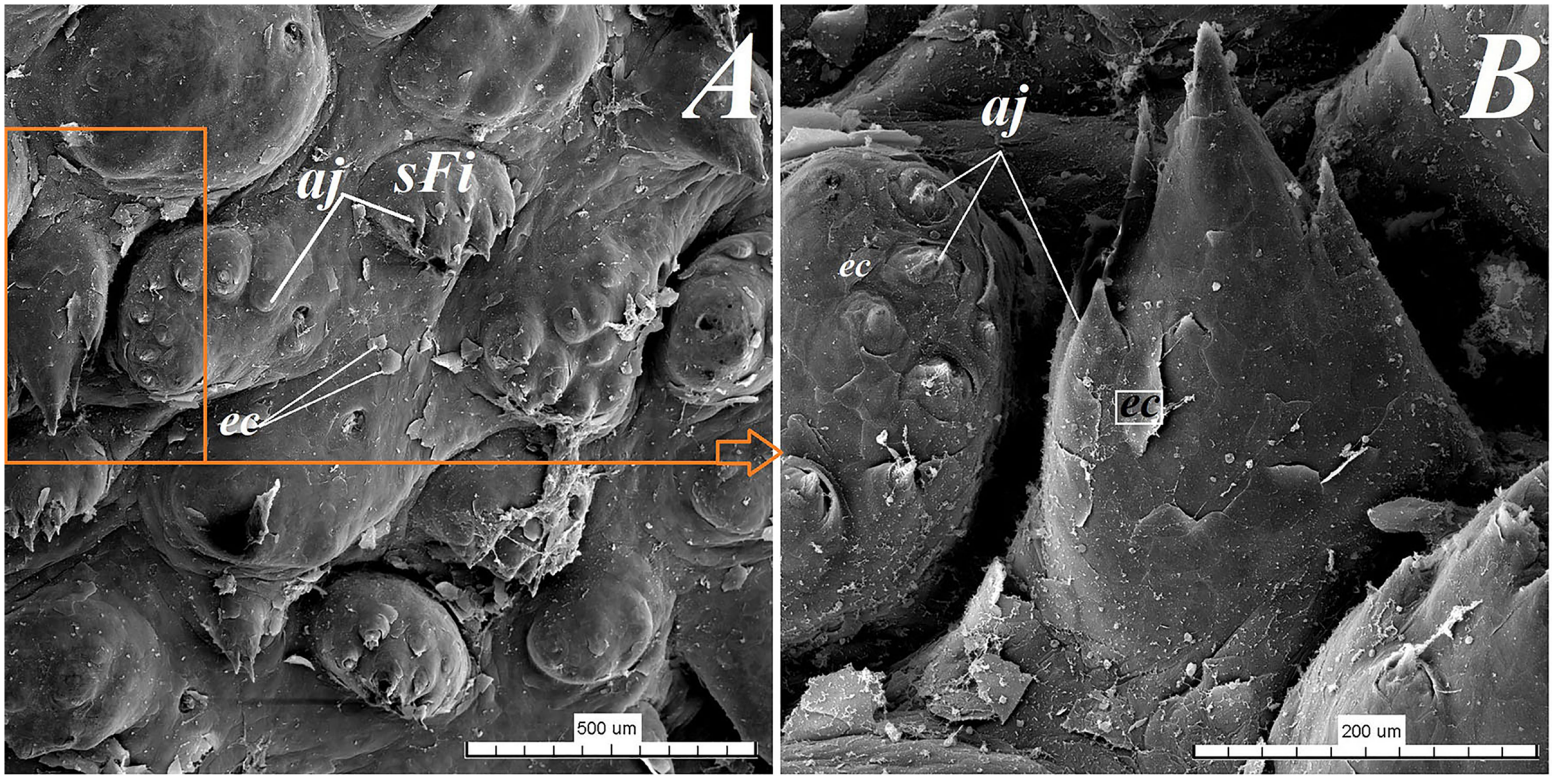
SEM analysis of the small filiform papillae from the area of the vallate papillae of the tongue of the red panda (*Ailurus fulgens f*.). (A) Small filiform papillae with several short secondary conical processes: SEM analysis. (B) Magnification of the two small filiform papillae with well defined exfoliated cells: SEM analysis. Bar = 500 µm (A); Bar = 200 µm (B). Abbreviations: aj, secondary processes; ec, exfoliated cell; sFi, small filiform papilla.

**Figure 10:**
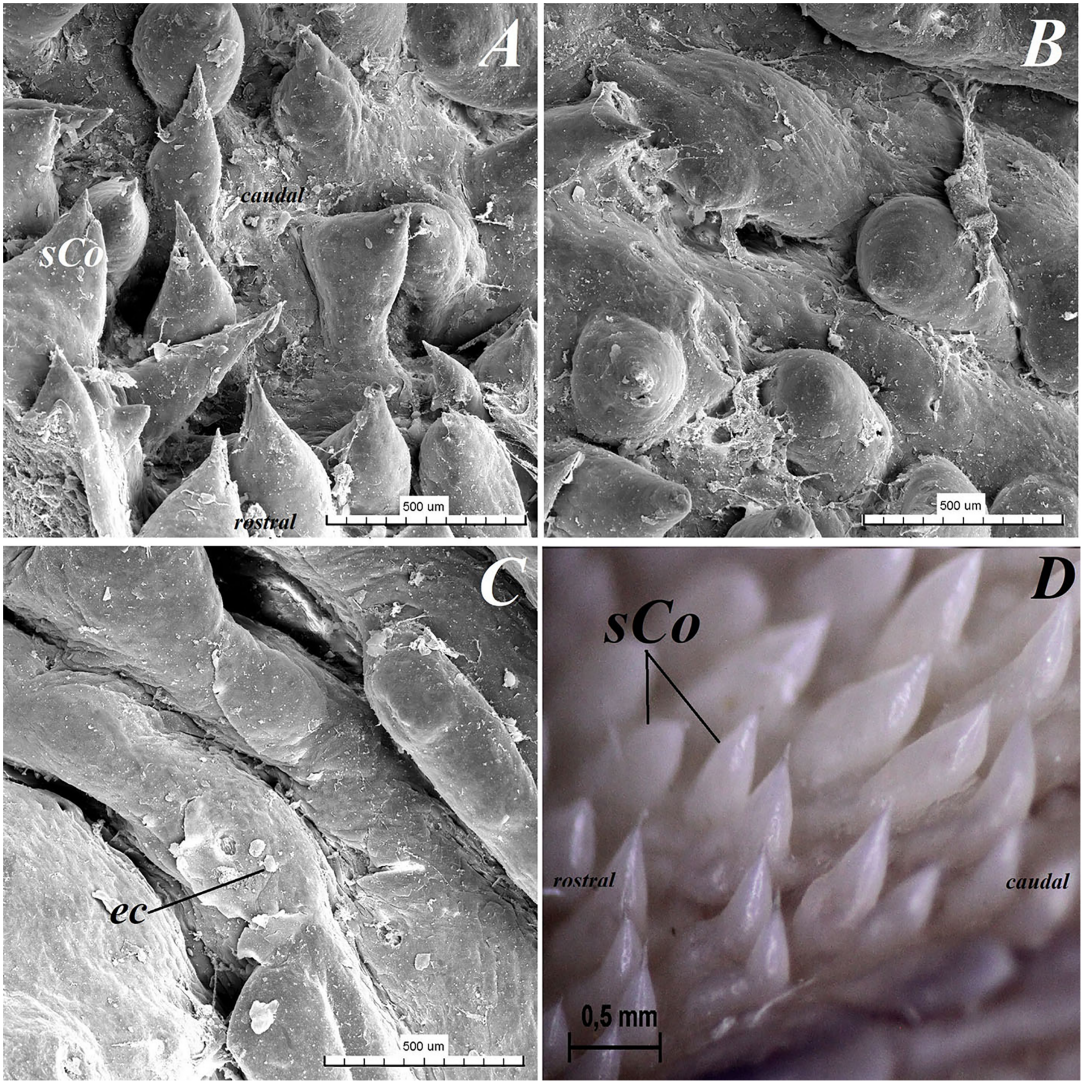
Stereoscopic and SEM analysis of the root of the tongue of the red panda (*Ailurus fulgens f*.). (A) Microstructure of the small conical papillae: SEM analysis. (B) Irregular dorsal surface of the root of tongue - SEM analysis. (C) Magnification of the irregular dorsal surface of the root of the tongue: SEM analysis. (D) Numerous small conical papillae: stereoscopic analysis. Bar = 500 µm (A–C); Bar = 0.5 mm (D). Abbreviations: ec, exfoliated cell; caudal, caudal orientation of the tongue; sCo, small conical papilla; rostral, rostral orientation of the tongue.

The height of vallate papillae was 869.33 ± 132.72 µm, while the width was 1,440.67 ± 126.53 µm. However, the *stratum corneum* of the epithelium of the vallate papillae formed a very thin layer (Fig. 7B). Within the connective tissue core of the vallate papillae, mucous acini and serous acini of lingual glands were also present (Figs. 7B and 7C). The SEM study showed that the surface of vallate papillae was irregular, however some of them had a smoother surface (Fig. 8B). The annular pads around of the vallate papillae also had an irregular surface (Fig. 8B). Around the vallate papillae, small filiform papillae with several short secondary conical processes were observed (Figs. 9A and 9B). The remaining dorsal section of the root of the tongue was irregular and covered with small conical papillae and openings of the lingual glands (Figs. 10A–10C). The small conical papillae were not secondarily divided (Figs. 10A and 10B).

### Lingual glands

**Figure 11:**
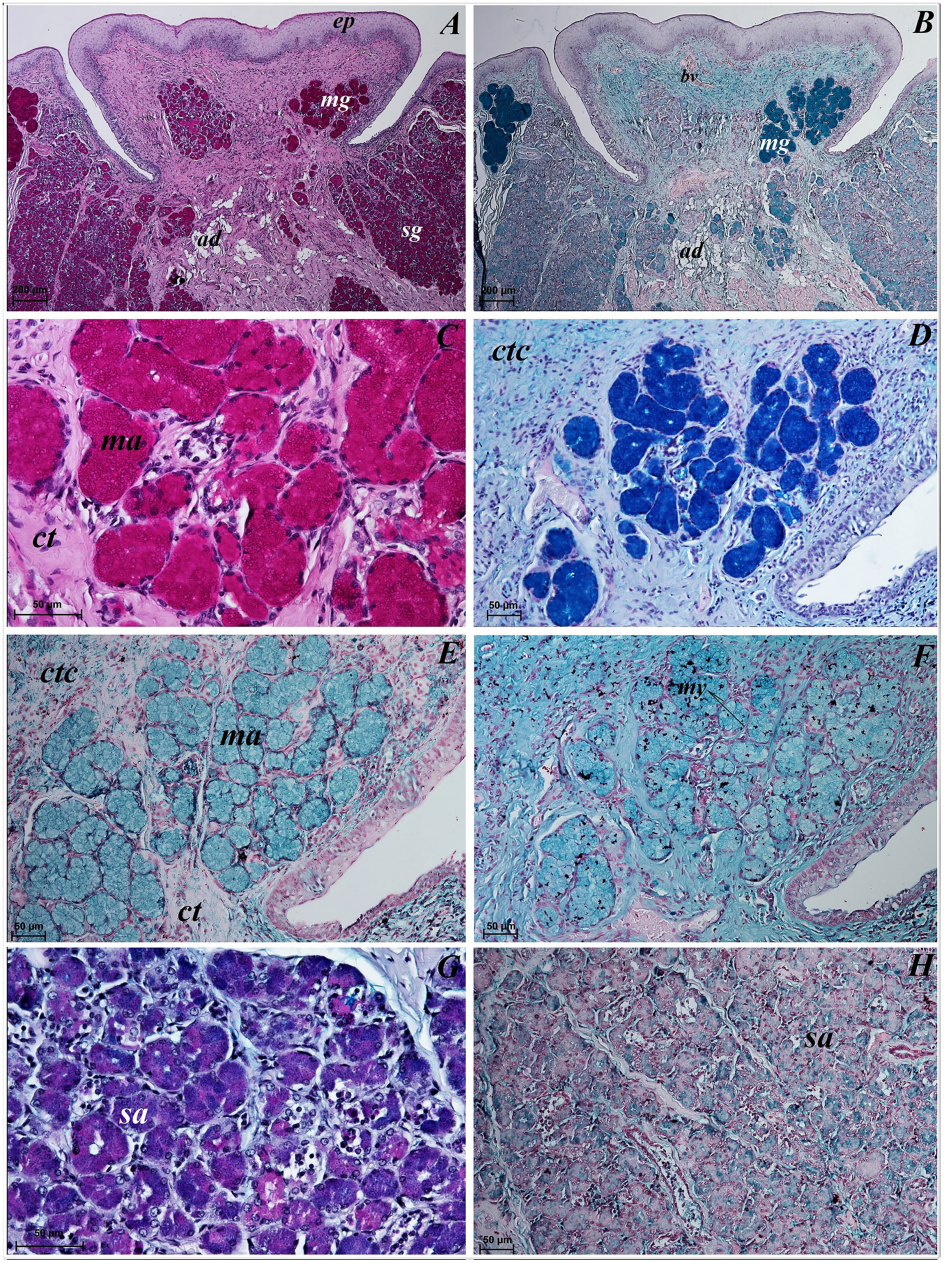
Histochemical visualization of the lingual glands concomitant to vallate papillae (gustatory gland) of the tongue of the red panda (*Ailurus fulgens f*.). (A) Strong positive reaction (+++) in mucous acini (rounded secretory units) (magenta) and weakly positive reaction (+) in serous acini. PAS staining. (B) Strong strong positive reaction (+++) in mucous acini (dark blue) and weakly positive reaction (+) in serous acini (light blue). AB pH2.5 staining. (C) Magnification of mucous acini with strong positive reaction (+++) (magenta). PAS staining. (D) Magnification of mucous acini with strong positive reaction (+++) (dark blue). PAS-AB pH2.5 staining. (E) Positive reaction (++) (blue) in mucous acini. HDI staining. (F) positive reaction (++) (blue) in mucous acini. AB pH1.0 staining. (G) Positive reaction (++) (magenta) in serous acini (rounded secretory units) and positive reaction (++) - dark blue color in some of cells. PAS-AB pH2.5 staining. (H) weakly positive reaction (+) – light blue color in serous acini. HDI staining. Bar = 200 µm (A,B); Bar = 50 µm (C–H). Abbreviations: ad, adipose cells. bv, blood vessel. ep, epithelium. ct, connective tissue. ma, mucous acini; mg, mucoserous glands with dominant mucous acini (rounded secretory units); my, myoepithelial cells; sa, serous acini; sg, serous glands.

Lingual glands in *Ailurus fulgens f*. were located directly below vallate papillae, and they were predominantly serous glands, whereas within the root of the tongue posterior mixed (mucoserous) lingual glands were predominant. Interestingly, mucous acini were also observed in the connective tissue core of the vallate papillae (Figs. 11A–11D), therefore not only serous glands were located within these papillae. Within the connective tissue below vallate papillae serous acini were predominant (Figs. 11A and 11B). The PAS staining of vallate papillae revealed strong positive reaction in mucous acini (rounded secretory units), while weakly positive reaction was observed in serous acini (Figs. 11A and 11C) which confirms the presence of neutral glycoconjugates in mucous acini. The AB pH 2.5 staining confirmed strong positive reaction in mucous acini and weakly positive reaction in serous acini (Fig. 11B). The PAS-AB pH 2.5 staining showed strong positive reaction (dark blue) in mucous acini (Fig. 11D), while positive reaction (magenta) in serous acini (rounded secretory units) and positive reaction-dark blue in some of cells (Fig. 11G), which confirms the presence of secretion containing combinations of both acidic and neutral glycoconjugates. The HDI staining showed positive reaction in mucous acini and weakly positive reaction in serous acini (Figs. 11E and 11H). The AB pH 1.0 staining showed positive reaction in mucous acini (Fig. 11F), which confirms the presence of sulphated glycoconjugates. Numerous openings of lingual glands were visible on the surface of the roof of the tongue.

**Figure 12:**
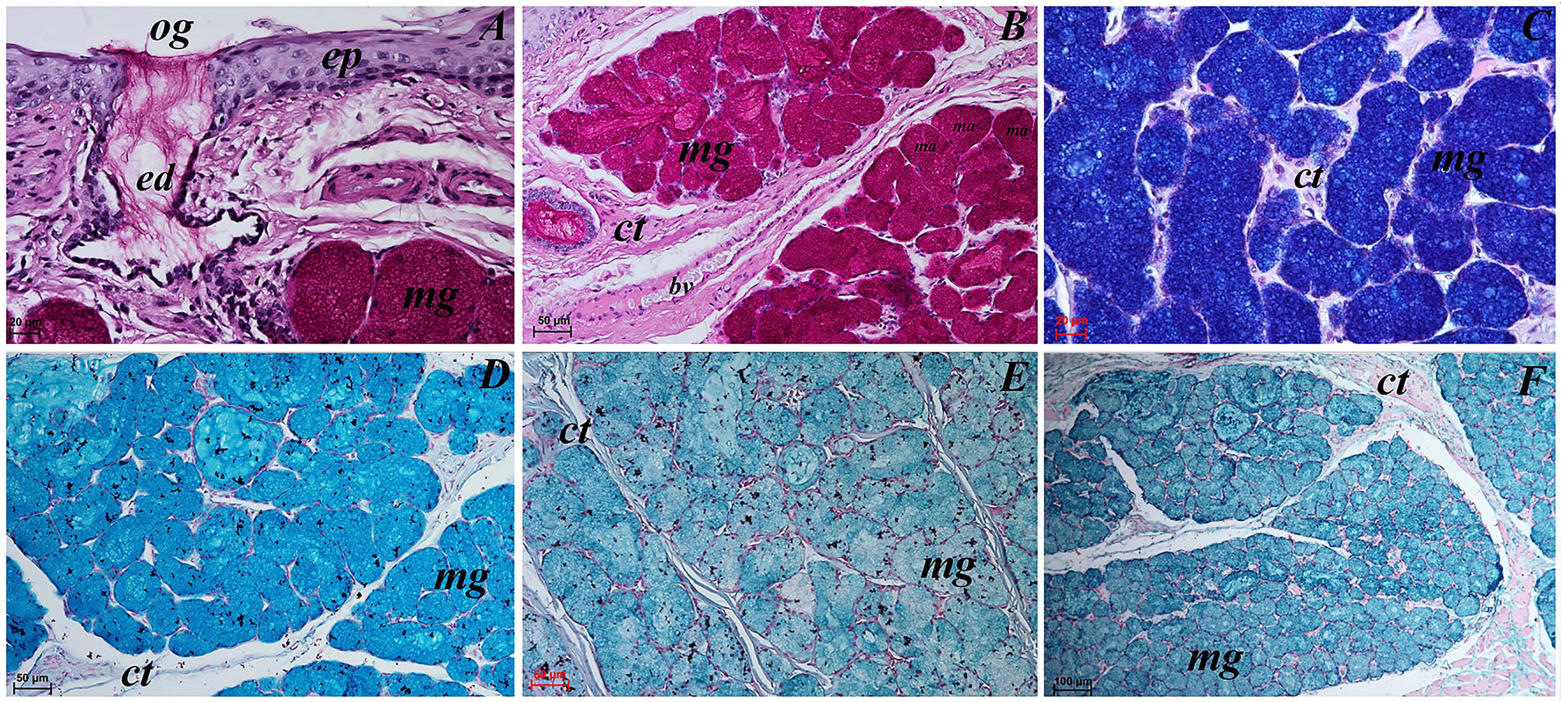
Histochemical visualization of the posterior lingual glands of the tongue of the red panda (*Ailurus fulgens f*.). (A) Magnification of the excretory duct. PAS staining. (B) Mucoserous glands (with dominant of mucous acini). Strong positive reaction (+++) in mucous acini. PAS staining. (C) Strong positive reaction (+++) – dark blue color in mucous acini. PAS-AB pH2.5 staining. (D) Strong positive reaction (+++) – blue color in mucous acini. AB pH2.5 staining. (E) positive reaction (++) (blue color) in mucous acini. AB pH1.0 staining. (F) Strong positive reaction (+++) (blue color) in mucous acini. HDI staining. Bar = 20 µm (A and C); Bar = 50 µm (B, D and E); Bar = 100 µm (F). Abbreviations: bv, blood vessel; ed, excretory duct; ep, epithelium; ct, connective tissue; ma, mucous acini (round secretory units); mg, mucoserous glands with dominant mucous acini; og, opening of the excretory duct.

Mucous units were prevalent within the area of posterior lingual glands. A single excretory duct had a broad light and was lined with epithelium composed of two layers of cells (Fig. 12A). Stratified squamous epithelium was observed by the opening of the excretory duct. The excretory duct was observed to be filled with secretions of posterior lingual glands (Fig. 12A).

The PAS staining showed strong positive reaction (magenta) in mucous acini (Fig. 12B). The PAS-AB pH 2.5 staining showed strong positive reaction in mucous acini (Fig. 12C) and AB pH 2.5 staining showed strong positive reaction (blue) in mucous acini (Fig. 12D). The AB pH 1.0 staining showed positive reaction in mucous acini. The HDI staining showed strong positive reaction in mucous acini, which confirms the presence of sulfated acid mucopolysaccharides or carboxylated acid mucopolysaccharides (Fig. 12F). The secretion of the posterior lingual glands was mucoserous.

## Discussion

The studies to date have paid much attention to the examination of the microstructure of lingual surface in mammals using various scientific methods. Results of many previous studies contribute to the development of comparative anatomy, and may also be included in clinical trials or veterinary surgery. Proper veterinary management of non-domestic species is a big challenge for veterinarians—among others—working in zoological gardens. Therefore, from this standpoint, a detailed knowledge of similarities and differences both in structure and function of particular organs (functional anatomy) is of paramount importance. Likewise, a detailed knowledge of the functional anatomy of digestive tract is linked to appropriate selection of food for captive animals. Although the microstructure of lingual surface was analysed in many different species of mammals (examples include the study of Kubota & Iwamoto, 1967; Emura et al., 2000, 2004; Iwasaki, 2002; Yoshimura, Shindoh & Kobayashi, 2002; Yoshimura et al., 2007; Pastor et al., 2011; Erdoğan et al., 2018; Iwasaki et al., 2019; Goździewska-Harłajczuk et al., 2016, 2020, Čížek et al., 2020), comparably with the results of other studies the detailed analysis of the lingual structure in *Ailurus fulgens f*. allowed us to conclude that in this endemic species there are features typical for the tongue related to the number and structure of the lingual papillae, and the presence of lyssa is another of these characteristic features.

The macroscopic study of the tongue in *Ailurus fulgens f*. showed that the apex of the tongue was rounded, similarly to the majority of species in the Carnivora order, including some species in the Caniformia suborder, such as—for instance—the wolf (Haligur, Ozkadif & Alan, 2019), the raccoon dog *Nyctereutes procyonoides* or the fox (Emura et al., 2006). A rounded apex, similarly as in red panda, was found in members of the Ursidae family, such as the Asian black bear *Ursus thibetanus* (Emura et al., 2001, Pastor et al., 2011), the American black bear *Ursus americanus*, the spectacled bear *Tremarctos ornatus*, the Malayan sun bear *Helarctos malayanus* (Pastor et al., 2011), the giant panda *Ailuropoda melanoleuca* (Pastor, Barbosa & De Paz, 2008, the polar bear *Ursus maritimus* (Emura, Sugiyama & Kusuda, 2017. However, a different feature was the bifurcated apex of the tongue in the Caniformia suborder, Phocidae family, namely in the spotted seal *Phoca largha* (Yoshimura et al., 2007). Furthermore, the difference in the dorsal surface of the tongue was the presence of median groove, which was not visible in *Ailurus fulgens f*., but in the wolf (Haligur, Ozkadif & Alan, 2019) was well-pronounced and elongated. On the other hand, in the Crab-eating racoon *Procyon cancrivorus* from the Procyonidae family, the median groove was hardly visible (Correa et al., 2012), and was also hardly visible on the apex of the tongue in the Northern racoon *Procyon lotor* (Miyawaki et al., 2020). Unlike *Ailurus fulgens f.*, in the Mustelidae family in the Asian short-clawed otter *Aonyx cinereus* the median groove was well-pronounced and elongated (Emura & Sugiyama, 2016). The presence of lingual prominence was not shown in *Ailurus fulgens f*., as in the members of the Procyonidae family: the Northern raccoon *Procyon lotor* (Miyawaki et al., 2020) and the crab-eating racoon (Correa et al., 2012), or in the Ursidae family: the Asian black bear *Ursus thibetanus* (Emura et al., 2001, Pastor et al., 2011), the American black bear *Ursus americanus*, the spectacled bear *Tremarctos ornatus*, the Malayan sun bear *Helarctos malayanus* (Pastor et al., 2011); in the giant panda *Ailuropoda melanoleuca* not very well-pronounced median groove was observed (Pastor, Barbosa & De Paz, 2008, the polar bear *Ursus maritimus* (Emura, Sugiyama & Kusuda, 2017. On the dorsal surface of the tongue in the red panda, 2 types of mechanical papillae and 2 types of gustatory papillae were observed. The biggest difference was in filiform papillae and conical papillae with respect to various members of the Caniformia suborders in the Carnivora order. Moreover, the number of vallate papillae was variable, which is consistent with the results of many earlier analyses of the microstructure of the lingual surface by other authors.

In the present study, the filiform papillae on the apex and body of the tongue in the *Ailurus fulgens f*. had different size of the main papillae and the filiform papillae on the apex had a few secondary papillae, which is comparable to the previous results of the SEM study of filiform papillae in the red panda by Emura, Okumura & Chen (2009). In contrast to the red panda, the tip of filiform papillae in the wolf *Canis lupus* had 1–4 bifurcations (Haligur, Ozkadif & Alan, 2019). However, in the Ursidae family—the Asian black bear *Ursus thibetanus* (Emura et al., 2001, Pastor et al., 2011), the American black bear *Ursus americanus*, the spectacled bear *Tremarctos ornatus*, the Malayan sun bear *Helarctos malayanus* (Pastor et al., 2011), the giant panda *Ailuropoda melanoleuca*—filiform papillae were multifilamentous with 3–13 secondary projections, or so called crowned filiform papillae were observed (Pastor, Barbosa & De Paz, 2008.

The typical filliform papillae shaped differently than the filiform papillae in the red panda were also observed in the Mustelidae family in the least weasel *Mustela nivalis*, whose filiform papillae on the apex had 10 secondary processes, whereas on the body of the tongue they had leaf-like shape (El-Bakary & Emura, 2016). Conical papillae were observed as small conical papillae in the *Ailurus fulgens f*., whereas in other species from the Caniformia suborders they were better pronounced, especially on the surface of root of the tongue. In the members of the Procyonidae family, in the Northern racoon *Procyon lotor* large conical papillae on the root were observed (Miyawaki et al., 2020), whereas in the crab-eating raccoon these papillae were observed also on the body and root of the tongue (Correa et al., 2012), in contrast to the red panda. Also in the Ursidae family in the Asian black bear *Ursus thibetanus* (Emura et al., 2001, Pastor et al., 2011), the American black bear *Ursus americanus*, the spectacled bear *Tremarctos ornatus*, the Malayan sun bear *Helarctos malayanus* (Pastor et al., 2011), the giant panda *Ailuropoda melanoleuca*, conical papillae were located caudally to the vallate papillae (Pastor, Barbosa & De Paz, 2008.

Fungiform papillae in *Ailurus fulgens f*. were essentially comparable to the shape of this kind of lingual papilla in the majority of the Carnivora. Mainly, the difference was the number of taste buds and the size of fungiform papillae. Mostly round in shape fungiform papilla in the red panda were similar to these papillae in the wolf (Haligur, Ozkadif & Alan, 2019). Contrary to the red panda, in the Mustelidae family in the least weasel *Mustela nivalis* few fungiform papillae were observed (El-Bakary & Emura, 2016). Similarly as in the family Ailuridae, fungiform papillae in the Ursidae family were round or oval in shape (Emura et al., 2001; Pastor, Barbosa & De Paz, 2008; Pastor et al., 2011; Emura, Sugiyama & Kusuda, 2017).

The 12–13 vallate papillae observed in *Ailurus fulgens f*. were distributed between the body and the root of the tongue, similarly as in the red panda in earlier studies (Emura, Okumura & Chen, 2009). However, the analysis of the number of vallate papillae in the red panda by Emura, Okumura & Chen (2009) showed the presence of only 11 vallate papillae. The results of the present and previous studies indicate that the difference in the number of these gustatory papillae may be individually variable. By comparison to red panda, significantly fewer vallate papillae are present in the wolf *Canis lupus*, a member of the Canidae family. In the wolf, 2 vallate papillae were found on both sides between the body and root of the tongue. Furthermore, on the uneven surface of these vallate papillae, 10–14 circular structure were observed in a SEM study (Haligur, Ozkadif & Alan, 2019), which was not found in *Ailurus fulgens f*. In the racoon dog *Nyctereutes procyonoides* and the fox *Vulpes vulpes japonica* (Emura et al., 2006), the bush dog *Speothos venaticus* (Emura et al., 2000) or the black-backed jackal numerous processes were observed on the surface of vallate papillae (Emura & Sugiyama, 2014).

A similar number of vallate papillae as in the red panda was found in some members of the Ursidae family, namely 9–14 (Emura et al., 2001; Pastor et al., 2011), whereas in the giant panda *Ailuropoda melanoleuca* 11 of them distributed either singly or in pairs next to each other were observed, along with numerous secondary papillae on their surface, visible in a SEM study (Pastor, Barbosa & De Paz, 2008). By comparison to the red panda, fewer vallate papillae were identified in the Mustelidae family: in the least weasel *Mustela nivalis* only 4 vallate papillae with central groove were found (El-Bakary & Emura, 2016), and only 4 vallate papillae in Japanese marten *Martes melampus* were found (Emura, Okumura & Chen, 2007), unlike in the Ailuridae family, and furthermore, in the Asian short-clawed otter *Aonyx cinereus* numerous secondary processes on the surface of vallate papillae were revealed (Emura & Sugiyama, 2016). Also, the presence of 7 vallate papillae distributed in “V” shape was characteristic of the Japanese badger *Meles anakuma* (Yoshimura, Shindo & Kageyama, 2009). The distribution of vallate papillae in “V” shape observed in the red panda was also typical of the crab-eating racoon *Procyon cancrivorus* from the Procyonidae family, in which 5 pairs of vallate papillae were revealed (Correa et al., 2012), whereas in the Northern racoon *Procyon lotor* 8 vallate papillae arranged in “V” shape were revealed (Miyawaki et al., 2020).

In the present study of the tongue of *Ailurus fulgens f*., foliate papillae were not observed, which was also demonstrated by previous macroscopic and SEM studies of the tongue in the red panda (Emura, Okumura & Chen, 2009. This is a feature that distinguishes the red panda from the members of the Caniformia, in which this type of lingual papillae occurs, such as wolf *Canis lupus*, in which foliate papillae were observed on both sides of the root of the tongue as four parallel leaves (Haligur, Ozkadif & Alan, 2019), or in the racoon dog *Nyctereutes procyonoides* and fox (Emura et al., 2006). The lateral region of the tongue root in the Procyonidae family was completely different from that in the red panda: in the Crab-eating racoon *Procyon cancrivorus*, in the area where foliate papillae should be located, three round papillae resembling vallate papillae were observed (Correa et al., 2012). In the Northern racoon *Procyon lotor*, weak fold-like foliate papillae were observed (Miyawaki et al., 2020), like in the red panda. In some members of the Mustelidae family, for instance the Japanese marten *Martes melampus*, some ridges and grooves of foliate papillae were observed (Emura, Okumura & Chen, 2007), contrary to *Ailurus fulgens f*.

Interestingly, unlike in the red panda, and also unusually, the presence of foliate papillae was found only in the American black bear *Ursus americanus* (among remaining members of the Ursidae family), and they had 5–6 folds (Pastor et al., 2011).

The present study revealed the presence of lyssa in *Ailurus fulgens f*. The shape of lyssa in the red panda was elongated and was comparable to the lyssa in the dog (Shoeib, Rizk & Hassanin, 2014). The color of lyssa was also similar to that in the dog, but differed from the lyssa in the cat, whose lyssa is more yellow (Shoeib, Rizk & Hassanin, 2014). A feature typical of lyssa in *Ailurus fulgens f*. was that it was surrounded with a connective tissue capsule, like in other species from the Carnivora order (Shoeib, Rizk & Hassanin, 2014).

Also in the red panda, like in the dog (Besoluk, Eken & Sur, 2006; Shoeib, Rizk & Hassanin, 2014, Sultana et al., 2017), lyssa was predominantly formed of adipose tissue. Its presence was also revealed in the Procyanide family in the crab-eating racoon (Correa et al., 2012). Although the role of lyssa has not been clearly determined so far, an earlier study of this structure in a primate, namely Sunda slow loris (*Nycticebus coucang*) confirmed the presence of simple sensory nerves in the tissue forming lyssa, and it probably also plays a role of a receptive lingual organ (Kubota & Iwamoto, 1967; Shoeib, Rizk & Hassanin, 2014). Furthermore, the existence of sex-specific differences in the structure of lyssa is not excluded, which should be studied on a larger number of tongues in the future.

Lingual glands observed in *Ailurus fulgens f*. among minor salivary glands were characterized by prevalence of mucous secretory units. Interestingly, lingual glands were also present in the connective tissue core of vallate papillae, which is a feature unique for this member of the Ailuridae family. A detailed analysis showed that the secretions were predominantly mucoserous.

Another aspect, apart from the comparative studies of the anatomy of the tongue in different animal species, is the reference of its structure to the type of food consumed (Iwasaki, 2002). In the case of the red panda, which is a highly selective forager, the diet is dominated by leaves, mainly young leaves and shoots of bamboo (Pradhan, Saha & Khan, 2001; Wei & Zhang, 2011a, 2011b; Panthi et al., 2012; Thapa & Basnet, 2015). The dominance of bamboo in the diet may be related to the fact that low nutrient content may help the poor digestive system of red panda (Thapa & Basnet, 2015). The lack of strongly developed mechanical conical papillae in *Ailurus fulgens f*. on the tongue surface, unlike, for example, mechanical lingual papillae in herbivores, probably may be related to the type of plant food that is particularly dominant in red panda. Therefore, the mucosa covering the tongue is not exposed to mechanical damage to the same extent as in herbivores, which also consume harder plant food.

The results of our research expand knowledge not only about the anatomy of the tongue in *Ailurus fulgens f*., but the results of these studies may be useful in veterinary medicine, especially for veterinarians specializing in working with exotic animals in veterinary clinics in zoos and people dealing with wildlife conservation in the case of such a valuable species like the red panda. Knowledge of the correct macroscopic and histological structure is extremely important from the point of view in veterinary surgery. Additionally, the results of the present study will form a basis for comparative anatomical studies of the biodiversity of endemic species and at the same time comparative studies in species where the diet is highly specialized as in *Ailurus fulgens f*. compared to other representatives phylogenetically related to the red panda. To summarizing, the results of our research refer not only to the basic anatomy of the tongue but may have a wider scope and support the above-mentioned other research areas.

## Conclusions

The study of the two tongues of *Ailurus fulgens f*. revealed that the presence of lyssa is a characteristic feature of the tongue of *Ailurus fulgens f.*, and also a feature comparable to other representatives of the Carnivora order. However, the number of vallate papillae was individually variable. Furthermore, the existence of further differences between the male and the female of the study species cannot be excluded, as well as possible differences in the number of gustatory papillae between *Ailurus fulgens f*. and *Ailurus fulgens s*. Therefore the present study is an initial analysis of features typical of the tongue of this representative of the Ailuridae family.

## Supplemental Information

10.7717/peerj.12559/supp-1 Supplemental Information 1 Surface of the tongues of red pandas. The figure is showing dorsal and ventral surface of the tongue from adult red panda male 1 (*Ailurus fulgens f*.) and vallate papillae area of red panda male 2 without any measurements or letters. These both tongues were used for detailed stereoscopic, LM and SEM analyses in our study.

## Additional Information and Declarations

### Data Availability

The following information was supplied regarding data availability: The raw data are available in the Supplemental File.
