# Supplementary figures and images for: The tongue of the red panda (Ailurus fulgens fulgens Cuvier, 1825)—a stereoscopy, light microscopy and ultrastructural analysis
Source: PeerJ. 2021 Nov 25;9:e12559. doi: 10.7717/peerj.12559 (PMC8627657; doi:10.7717/peerj.12559)

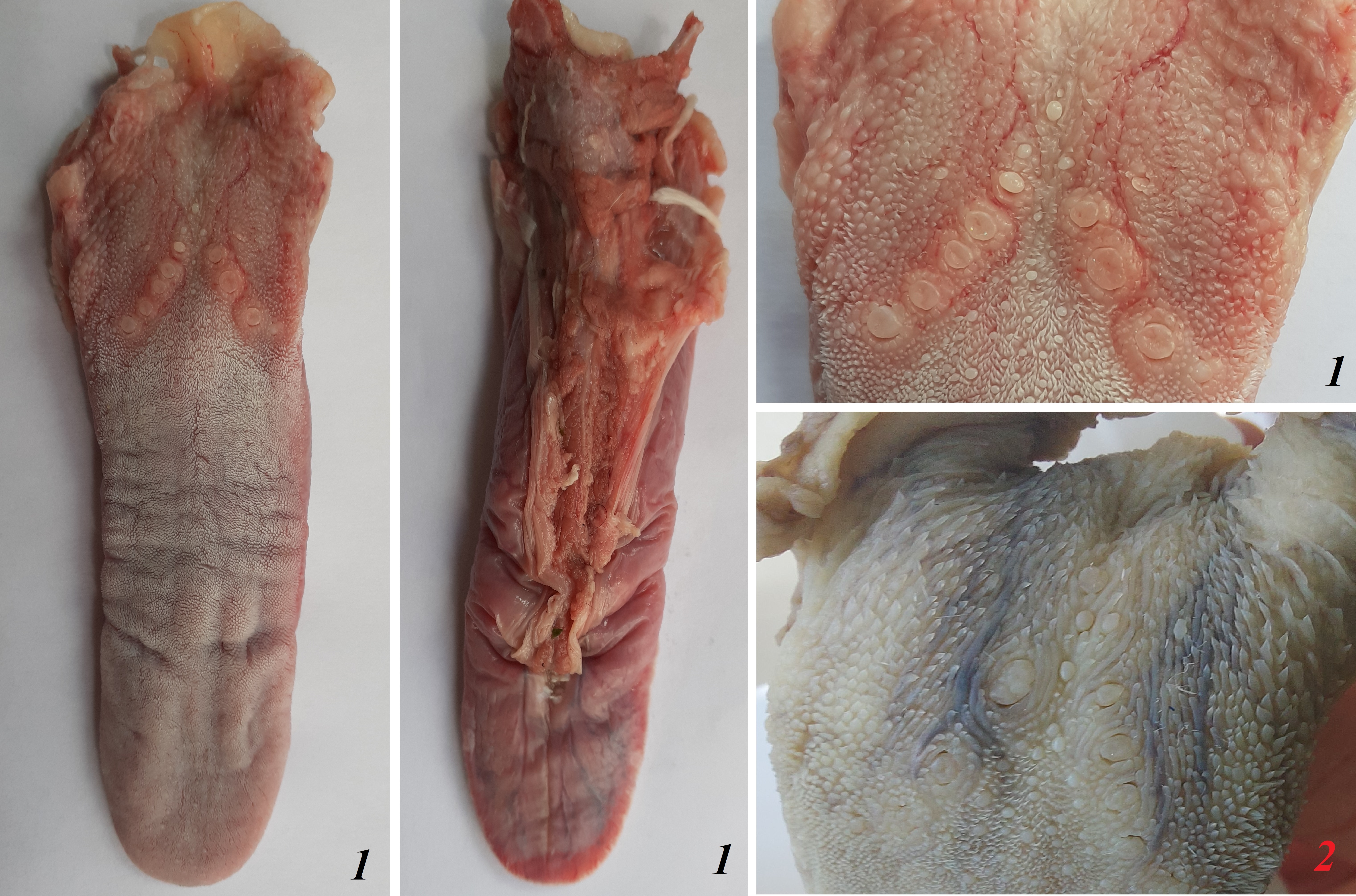

Supplement: Supplemental Information 1 — The figure is showing dorsal and ventral surface of the tongue from adult red panda male 1 (Ailurus fulgens f.) and vallate papillae area of red panda male 2 without any measurements or letters. These both tongues were used for detailed stereoscopic, LM and SEM analyses in our study. [file peerj-09-12559-s001.jpg]
